# Serum Concentrations of Trace Elements in Patients with Tuberculosis and Its Association with Treatment Outcome

**DOI:** 10.3390/nu7075263

**Published:** 2015-07-21

**Authors:** Rihwa Choi, Hyoung-Tae Kim, Yaeji Lim, Min-Ji Kim, O Jung Kwon, Kyeongman Jeon, Hye Yun Park, Byeong-Ho Jeong, Won-Jung Koh, Soo-Youn Lee

**Affiliations:** 1 Department of Laboratory Medicine and Genetics, Samsung Medical Center, Sungkyunkwan University School of Medicine, 81 Irwon-Ro, Gangnam-Gu, Seoul 135-710, Korea; E-Mails: rihwa.choi@samsung.com (R.C.); elbereth.kim@samsung.com (H.-T.K.); 2 Biostatistics and Clinical Epidemiology Center, Samsung Medical Center, 81 Irwon-Ro, Gangnam-Gu, Seoul 135-710, Korea; E-Mail: yaeji.lim@samsung.com; 3 Biostatistics team, Samsung Biomedical Research Institute, 81 Irwon-Ro, Gangnam-Gu, Seoul 135-710, Korea; E-Mail: minji93.kim@samsung.com; 4 Division of Pulmonary and Critical Care Medicine, Department of Medicine, Samsung Medical Center, Sungkyunkwan University School of Medicine, 81 Irwon-Ro, Gangnam-Gu, Seoul 135-710, Korea; E-Mails: ojung.kwon@samsung.com (O.J.K.); kyeongman.jeon@samsung.com (K.J.); kerrybe.park@samsung.com (H.Y.P.); bh82.jeong@samsung.com (B.-H.J.)

**Keywords:** tuberculosis, trace elements, inductively coupled plasma-mass spectrometry, Korea

## Abstract

Deficiencies in essential trace elements are associated with impaired immunity in tuberculosis infection. However, the trace element concentrations in the serum of Korean patients with tuberculosis have not yet been investigated. This study aimed to compare the serum trace element concentrations of Korean adult patients with tuberculosis with noninfected controls and to assess the impact of serum trace element concentration on clinical outcome after antituberculosis treatment. The serum concentrations of four trace elements in 141 consecutively recruited patients with tuberculosis and 79 controls were analyzed by inductively coupled plasma-mass spectrometry. Demographic characteristics were also analyzed. Serum cobalt and copper concentrations were significantly higher in patients with tuberculosis compared with controls, while zinc and selenium concentrations were significantly lower (*p* < 0.01). Moreover, serum selenium and zinc concentrations were positively correlated (ρ = 0.41, *p* < 0.05). A high serum copper concentration was associated with a worse clinical outcome, as assessed after one month of antituberculosis therapy. Specifically, culture-positive patients had higher serum copper concentrations than culture-negative patients (*p* < 0.05). Patients with tuberculosis had altered serum trace element concentrations. Further research is needed to elucidate the roles of individual trace elements and to determine their clinical impact on patients with tuberculosis.

## 1. Introduction 

Although treatments are available, tuberculosis still remains a leading cause of death worldwide [1]. The revised estimate in the latest 2014 World Health Organization global tuberculosis report states that almost half a million more cases of tuberculosis occurred worldwide compared with the 2013 estimate; moreover, 1.5 million people died (up from 1.3 million in 2012) out of the estimated nine million people who developed tuberculosis in 2013 [1]. Tuberculosis and malnutrition are linked in a complex relationship. Specifically, tuberculosis infection may cause malnutrition through increased metabolic demands and decreased nutrient intake, while nutritional deficiencies may worsen the disease or delay recovery by inhibiting important immune functions [2]. People with tuberculosis are often malnourished, and malnourished people are also at a higher risk of developing tuberculosis since their immune systems are weakened [2]. Micronutrient environments are key contributors to immune function and cytokine kinetics. Thus, such environments have been increasingly suggested to play an essential role in the individual response to infectious diseases [3]. Trace elements that are essential micronutrients are known to play a variety of important roles, including as structural components of vitamins (e.g., cobalt); as co-factors in metalloenzymes, such as glutathione peroxidase (e.g., selenium); as catalytic components of numerous enzymes (e.g., zinc and copper); and as structural components of other proteins important for immune reactions (also zinc and copper) [4,5]. Due to their immunomodulatory functions, trace elements have been hypothesized to influence the susceptibility of the human body to the courses and outcomes of various infections [3]. Deficiencies in various essential trace elements have been associated with decreased immunity against tuberculosis infection; moreover, trace elements are believed to have an impact on clinical outcome and are thus related to disease control [2]. Altered profiles of trace elements have been reported in different populations of patients with tuberculosis [6,7,8]. Trace element concentrations in serum depend not only on external factors, including micronutrient status, but also on many internal factors; profiles are believed to be influenced by the host physiology, pathogen physiology, and host diet [4]. However, the availability of these trace elements for digestion, absorption, and utilization, in addition to their clinical impacts on patient outcomes, are still under investigation [6,7,8]. Moreover, no study has yet obtained a reliable estimate of the trace element concentrations in the serum of Korean patients with tuberculosis.

Among the various techniques used to analyze trace element concentrations in human biological fluids (e.g., serum and plasma) and tissues, inductively coupled plasma-mass spectrometry (ICP-MS) is widely used. This technique has been used to compare normal with disease conditions, to detect potentially toxic metals, and to diagnose trace element deficiency states and trace element-related diseases. Moreover, ICP-MS has the advantage of being able to screen multiple elements with high sensitivity [9].

Therefore, this study aimed to investigate the serum concentrations of four trace elements (cobalt, copper, zinc, and selenium) in Korean adult patients with tuberculosis and to compare these values with those of control patients (not infected with tuberculosis). Trace element concentrations were measured using a standard ICP-MS procedure, and potential associations between micronutrient concentrations and basal characteristics were assessed. Furthermore, the impact of trace element concentrations in serum on clinical outcomes after an intensive phase of antituberculosis therapy in patients with tuberculosis was assessed.

## 2. Methods

### 2.1. Study Populations, Diagnosis, and Treatment of Tuberculosis

Korean adult patients with tuberculosis who visited Samsung Medical Center, a tertiary referral hospital, were recruited consecutively between January 2014 and January 2015. All patients met the diagnostic criteria for pulmonary tuberculosis according to the guidelines of the American Thoracic Society [10]. Diagnosis was based on culture positivity from at least two separate expectorated sputum samples and culture positivity on bronchial wash or bronchoalveolar lavage fluids. Sputum smear tests and mycobacterial culture tests were performed according to standard methods [10]. The patients in the tuberculosis group were bacteriologically confirmed as infected with *Mycobacterium tuberculosis* (*M. tuberculosis*). Patients infected with multiple drug-resistant strains of *M. tuberculosis* and patients infected with nontuberculous mycobacteria were excluded after drug sensitivity tests or pathogen identification. Treatment response was determined by *M. tuberculosis* culture tests and sputum acid-fast bacilli (AFB) smears after one month of an established treatment course to assess the clinical impact of trace element concentrations in serum on treatment outcome. Patients who were culture-positive or smear-positive one month after the initiation of treatment were regarded as slow responders. Individuals for the control group were selected from people who visited the health promotion center for regular health checkups, did not exhibit any clinical symptoms or signs of illness, and volunteered for blood sampling. Inclusion criteria for the nontuberculosis controls were: (1) absence of cough, fever, or evidence of pulmonary disease at the time of enrollment; and (2) no prior diagnosis of tuberculosis disease and no known or suspected household contact with pulmonary tuberculosis in the past 2 years. All participants enrolled in this study were adults. Trace element concentrations were compared between patients with tuberculosis and noninfected controls and were also analyzed in the context of demographic characteristics and other serologic markers used to assess nutritional status. Body mass index (BMI), total protein, albumin, C-reactive protein (CRP), and total cholesterol concentrations were used to assess nutritional status. The concentrations of those markers were measured at the same time as the serum trace element concentrations.

### 2.2. Ethics Statement

Informed written consent was obtained from all participants. This study was approved by the Institutional Review Board of Samsung Medical Center (IRB No. SMC-2013-07-155-002).

### 2.3. Analytical Procedures

Blood samples were collected in plastic blood collection tubes designated for trace element analysis (BD Vacutainer Trace Element tube with potassium-EDTA, BD, Oxford, UK). Serum was separated from whole blood and aliquots were stored at −70 °C until trace element analysis. Serum samples were diluted 1:40 in a solution containing 2.5% (v/v) tetramethylammonium hydroxide, 1% (v/v) n-butanol, 0.001% (v/v) Triton X-100, and 1 μg/L gallium as an internal standard. The four trace elements (cobalt, copper, zinc, and selenium) were analyzed with a quadrupole inductively coupled plasma-mass spectrometer equipped with a concentric glass nebulizer and a cyclonic spray chamber. Serum trace element concentrations were determined by ICP-MS (7700x ICP-MS system, Agilent Technologies^®^, Santa Clara, CA, USA) as previously described but with minor modifications [11]. Two isotopes of each element were measured to ensure accurate results. National Institute of Standards and Technology (NIST)-traceable 10 mg/L and 1000 mg/L elemental standards were used for preparation of multielement calibration standards. Standards passed the calibration cutoff if their back-calculated concentrations were within ±15% of the nominal concentrations. Both intra- and inter-assay imprecision were <10% of the precision coefficient of variation. The accuracy of serum zinc, copper, and selenium measures was assured using the Proficiency Testing/Quality Management program of the Unites States College of American Pathologists (CAP) survey. The assay ranges were 0.01–100 µg/L for cobalt, 1.0–250 μg/dL for copper, 1.0–350 µg/dL for zinc, and 1.0–200 µg/L for selenium.

Serum total protein, albumin, aspartate aminotransferase (AST), alanine aminotransferase (ALT), alkaline phosphatase (ALP), total cholesterol concentrations, and CRP levels were measured on a Roche modular analyzer (Roche Diagnostics Corp., Indianapolis, IN, USA) as indicated in the manufacturer’s instructions.

### 2.4. Statistical Analysis

Data were analyzed using R (3.1.2, The R Foundation for Statistical Computing). The Shapiro–Wilk test was used to assess whether the data were normally distributed. Serum concentrations of the four trace elements in patients with tuberculosis were compared with those in control patients using Student’s *t*-test and the Wilcoxon rank sum test. Relationships among four trace elements were analyzed using Spearman correlation analysis. *p*-Values less than 0.05 were considered statistically significant.

## 3. Results

The demographic characteristics, nutritional statuses, serum chemistry results, and concentrations of the four trace elements for patients and controls are shown in Table 1, Table 2 and Table 3. In the control group, age, BMI, total serum proteins, total cholesterols, and selenium concentrations were normally distributed. In the tuberculosis patient group, BMI, total serum protein, total cholesterol, and selenium concentrations were normally distributed. BMI and total cholesterol were significantly higher in the control group, whereas the total protein, ALP, and CRP were significantly higher in the tuberculosis group (*p* < 0.05). No significant differences between the two groups were observed regarding any of the other parameters measured, including serum albumin concentration.

**Table 1:** Demographic characteristics and trace element concentrations in the control group without pulmonary tuberculosis (*n* = 79).

Parameter	Mean	SD	Median	25th ‰	75th ‰	Min	Max	*p*-Value ^*^
Age (years)	46.9	11.8	46.0	38.0	55.0	21.0	72.0	0.3076
BMI (kg/m^2^)	23.3	3.0	23.8	21.1	25.4	16.4	31.2	0.6420
Total protein (g/dL)	7.1	0.4	7.1	6.7	7.4	6.3	7.9	0.1241
Albumin (g/dL)	4.3	0.2	4.3	4.2	4.4	3.9	5.1	0.0064
Total cholesterol (mg/dL)	208	33	208	181	235	138	274	0.1688
AST (U/L)	23	9	21	17	26	9.0	55	0.0000 ^†^
ALT (U/L)	24	20	18	13	25	7	127	0.0000 ^†^
ALP (U/L)	61	17	58	48	71.0	34	125	0.0012
CRP (mg/dL)	0.1	0.0	0.0	0.0	0.1	0.0	0.3	0.0000 ^†^
Cobalt (µg/L)	0.23	0.20	0.15	0.12	0.24	0.07	1.20	0.0000 ^†^
Copper (µg/dL)	85	17	83	74	90	54	189	0.0000 ^†^
Zinc (µg/dL)	107	21	104	91	116	67	178	0.0060
Selenium (µg/L)	114	15	113	102	123	84	157	0.3013

SD, standard deviation; Min, minimum value; Max, maximum value; BMI, body mass index; AST, aspartate aminotransferase; ALT, alanine aminotransferase; ALP, alkaline phosphatase; CRP, C-reactive protein; ^*^ *p*-value determined using the Shapiro–Wilks test for normality; ^†^ *p*-value < 0.0001.

**Table 2:** Demographic characteristics and trace element concentrations in pulmonary tuberculosis patients (*n* = 135).

Parameter	Mean	SD	Median	25th ‰	75th ‰	Min	Max	*p*-Value ^*^
Age (years)	50.0	15.2	50.0	38.0	63.0	19.0	80.0	0.0052
BMI (kg/m^2^)	22.0	2.9	21.8	20.0	24.3	15.8	30.7	0.3659
Total protein (g/dL)	7.3	0.6	7.4	7.0	7.7	5.6	9.0	0.2572
Albumin (g/dL)	4.3	0.4	4.3	4.1	4.6	2.8	5.0	0.0007
Total cholesterol(mg/dL)	170	34	172	149	192	47	249	0.3821
AST (U/L)	29	36	23	19	29	11	416	0.0000 ^†^
ALT (U/L)	23	20	17	13	27	5	144	0.0000 ^†^
ALP (U/L)	72	31	67	56	80	9	289	0.0000 ^†^
CRP (mg/dL)	1.0	2.1	0.2	0.1	0.7	0.0	15	0.0000 ^†^
Cobalt (µg/L)	0.29	0.14	0.25	0.21	0.33	0.10	0.80	0.0000 ^†^
Copper (µg/dL)	122	34	115	102	139	65	294	0.0000 ^†^
Zinc (µg/dL)	67	13	66	59	73	30	128	0.0000 ^†^
Selenium (µg/L)	104	19	103	91	115	62	168	0.10028

SD, standard deviation; Min, minimum value; Max, maximum value; BMI, body mass index; AST, aspartate aminotransferase; ALT, alanine aminotransferase; ALP, alkaline phosphatase; CRP, C-reactive protein; ^*^ *p*-value determined using the Shapiro–Wilks test for normality; ^†^ *p*-value < 0.0001.

**Table 3:** Comparison of demographic characteristics and trace element concentrations of pulmonary tuberculosis patients (*n* = 135) and the control group without pulmonary tuberculosis (*n* = 79).

Parameter	Controls (*n* = 79)		TB (*n* = 135)		*p*-Value ^*^
	Mean	Median	Mean	Median	
Age (years)	46.9	46.0	50.0	50.0	0.1393 ^†^
Sex (*n*)					0.0678 ^‡^
Male (*n*)	44		92		
Female (*n*)	35		43		
BMI (kg/m^2^)	23.3	23.8	22.0	21.8	0.0017 ^§^
Total protein (g/dL)	7.1	7.1	7.3	7.4	0.0004 ^§^
Albumin (g/dL)	4.3	4.3	4.3	4.3	0.7824 ^†^
Total cholesterol (mg/dL)	208	208	170.2	172	0.0000 ^§ ¶^
AST (U/L)	23	21	29	23	0.0178 ^†^
ALT (U/L)	24	18	23	17	0.9781 ^†^
ALP (U/L)	61	58	72	67	0.0009 ^†^
CRP (mg/dL)	0.1	0.0	1.0	0.2	0.0000 ^† ¶^
Cobalt (µg/L)	0.23	0.15	0.29	0.25	0.0000 ^† ¶^
Copper (µg/dL)	85	83	122	115	0.0000 ^† ¶^
Zinc (µg/dL)	107	104	67	66	0.0000 ^† ¶^
Selenium (µg/L)	114	113	104	103	0.0000 ^§ ¶^

TB, tuberculosis patients; BMI, body mass index; AST, aspartate aminotransferase; ALT, alanine aminotransferase; ALP, alkaline phosphatase; CRP, C-reactive protein; ^*^ *p*-values from univariable analysis; ^†^ *p*-values from the Wilcoxon rank sum Test; ^‡^ *p*-values from the chi-square test; ^§^ *p*-values from the *t*-test; ^¶^ *p*-value < 0.0001.

**Figure 1:**
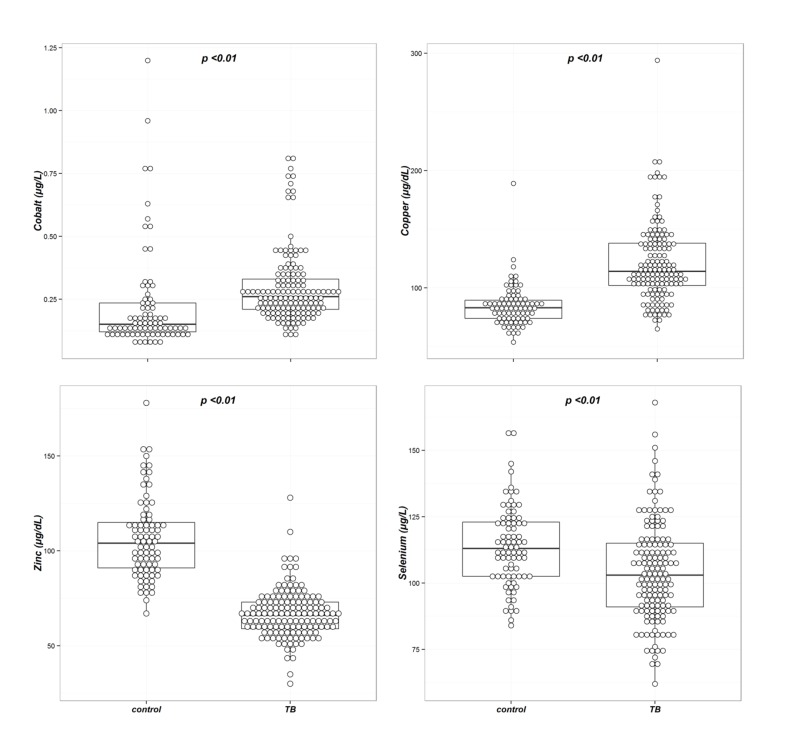
Box plots of the trace element concentrations in the tuberculosis and control groups. The concentrations of all four trace elements were significantly different between the two groups.

The concentrations of all four trace elements were significantly different between the two groups (Table 3 and Figure 1). In univariable analysis, the mean serum concentration ± SD (standard deviation) of selenium was significantly lower in the tuberculosis group compared with the control group (114 ± 15 *vs.* 104 ± 19 µg/L, *p* < 0.01). The median serum cobalt and copper concentrations were significantly higher in the tuberculosis group compared with the control group (0.15 *vs.* 0.25 µg/L, *p* < 0.01 for cobalt, and 83 *vs.* 115 µg/dL, *p* < 0.01 for copper, respectively); the median zinc concentration was significantly lower in the tuberculosis group (104 *vs.* 66 µg/dL, *p* < 0.01).

Multivariable analysis was performed to investigate factors associated with trace element concentrations; variables with a *p*-value of less than 0.05 in univariable analysis were included in multivariable analysis. Copper and zinc concentrations were log-transformed for multivariable analysis, the results of which are summarized in Table 4. In multivariable analysis, serum cobalt, copper, and zinc concentrations were significantly different between controls and tuberculosis patients (*p* < 0.01), while serum selenium concentrations were not (*p* = 0.14). Multivariable analysis revealed that BMI was associated with cobalt and zinc concentration, total protein, ALP, and CRP were associated with copper centration, and BMI, total protein, and total cholesterol were associated with selenium concentration (*p* < 0.05).

**Table 4:** Factors associated with four trace element concentrations: *p*-values for multivariable analysis.

Parameter	Cobalt	Copper ^*^	Zinc ^*^	Selenium
Presence of tuberculosis	0.0000 ^†^	0.0000 ^†^	0.0000 ^†^	0.1411
BMI	0.0000 ^†^	0.1800	0.0004	0.0003
Total protein	0.5739	0.0004	0.1641	0.0074
Total cholesterol	0.2103	0.6184	0.4125	0.0032
AST	0.0783	0.2198	0.2528	0.7848
ALP	0.2826	0.0115	0.0808	0.5724
CRP	0.0840	0.0000 ^†^	0.3127	0.1111

BMI, body mass index; AST, aspartate aminotransferase; ALP, alkaline phosphatase; CRP, C-reactive protein. Variables with a *p*-value of less than 0.05 in univariable analysis (in Table 3) were included in multivariable analysis. ^*^ Copper and zinc concentrations were log-transformed for multivariable analysis; ^†^ *p*-value < 0.0001.

**Figure 2:**
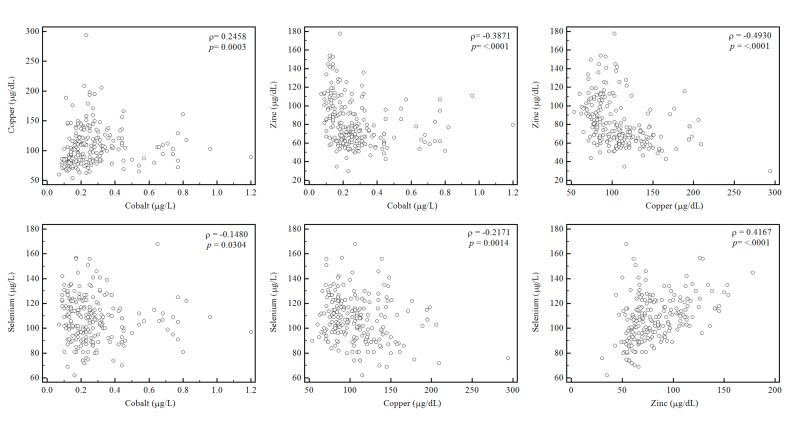
Correlations among concentrations of four trace elements. Serum copper and zinc showed a moderate negative correlation (ρ = −0.4930, *p* < 0.001) and serum zinc and selenium concentration showed a moderate positive correlation (ρ = 0.4167, *p* < 0.05).

Correlations among four trace elements (Spearman correlation analysis) demonstrated a negative relationship between serum zinc and copper concentrations (ρ = 0.49, *p* < 0.05), and a positive correlation between serum zinc and selenium concentrations (ρ = 0.41, *p* < 0.05, Figure 2). The analysis of the relationship between trace element concentrations and CRP level revealed a moderate positive correlation with copper (ρ = 0.41, *p* < 0.01), a weak negative correlation with zinc (ρ = −0.15, *p* = 0.02), and a weak negative correlation with selenium (ρ = −0.16, *p* < 0.01).

**Figure 3:**
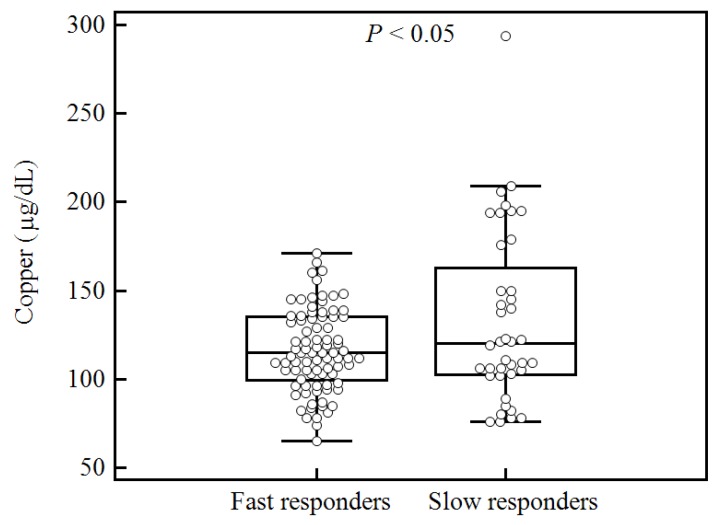
Comparison of serum copper levels at the time of diagnosis between fast responders and slow responders defined by the culture-positive and culture-negative results after one month of standard antituberculosis therapy.

We next determined whether trace element concentrations were associated with clinical outcomes. Patients with tuberculosis underwent antituberculosis treatment and follow-up smear tests. Trace element concentrations were then compared and examined for correlation with treatment outcomes. Among 141 patients with tuberculosis, 40 (28.4%) had positive follow-up culture tests or smear tests after one month of antituberculosis treatment. Of the four trace elements, only serum copper level (at the time of diagnosis) was correlated with treatment outcome; specifically, culture-positive patients tended to have higher serum copper concentrations (*p* < 0.05, Figure 3).

## 4. Discussion

In this study, we investigated the concentrations of four serum trace elements in healthy controls compared with tuberculosis patients. The serum trace element concentrations (mean ± SD) in this study were 0.23 ± 0.20 μg/L for cobalt, 85 ± 17 μg/L for copper, 107 ± 21 μg/dL for zinc, and 114 ± 15 μg/dL for selenium in controls without tuberculosis. Only a few studies have reported serum trace elements concentrations identified using ICP-MS in healthy Korean subjects. The serum cobalt concentration has been reported to range from 0.29 ± 0.15 μg/L in 51 healthy Korean subjects [12] to 0.47 ± 0.27 μg/L in 103 healthy subjects [11]. Serum copper and zinc concentrations had reported averages of 91.32 ± 21.65 μg/dL and 80.45 ± 15.08 μg/dL, respectively, in 103 healthy subjects [11]. The mean serum selenium concentration was 112.05 ± 30.42 μg/L in 100 healthy subjects [13] and 81.20 ± 31.47 μg/L in 103 healthy subjects [11]. The differences in trace element concentrations between previous studies and the present study could be related to variation in the age and sex distribution of participants [11,13].

This study found that the serum zinc and selenium concentrations were significantly lower in patients with tuberculosis compared with control patients, while the copper concentrations were higher. These findings are comparable with those of previous studies performed in Ethiopia, Italy, and Indonesia [6,7,8]. Furthermore, the present study identified a positive correlation between the serum zinc and selenium concentrations. Zinc has been implicated in antioxidant defense via the antioxidant metalloenzyme copper-zinc superoxide dismutase. Specifically, zinc binds to the redox active sites and prevents the binding of more damaging metals. Zinc also regulates metallothioneins, which have roles in free radical scavenging and inflammatory processes [14]. Selenium has direct antioxidant activity as a result of its incorporation into selenoproteins, such as glutathione peroxidases, thioredoxin reductase, and some isoforms of methionine sulfoxide reductase [15]. Zinc and selenium are both essential for cell-mediated and humoral immunity, and their statuses have been shown to affect the function of adaptive and innate immune cells [16,17,18]. Zinc and selenium deficiencies can increase susceptibility to tuberculosis infection and leave individuals vulnerable to oxidative stress. Although a recent systematic review reported that the plasma concentrations of zinc and selenium can be improved by supplementation during the early stages of tuberculosis treatment, a consistent benefit from supplementation on tuberculosis treatment outcome and/or nutritional recovery has not been demonstrated [2]. Future research is needed to determine the extent to which these trace elements impact clinical outcomes and also to establish whether the routine administration of supplements results in better tuberculosis treatment outcomes and/or improved life quality.

Copper is involved in the natural defense system against reactive oxygen species [19]. Moreover, iron and copper are interlinked via ceruloplasmins, which exhibit ferroxidase activity and are suitable for assessing free radical activity [20]. The maintenance of copper homeostasis, which involves redistribution and mobilization, has been reported to be key in the immune response to tuberculosis infection [20]. In this study, the mean serum copper concentration was significantly higher in patients with tuberculosis compared with control patients. This finding is comparable with those of previous studies performed in Ethiopia, Italy, and Canada [6,7,21]. The negative correlation between copper and zinc concentrations observed in this study was comparable to the results presented in previous literature, and could be explained by the ability of zinc ions to block copper absorption, possibly by formation of intestinal metallothionein, which strongly binds copper [4]. Among the four trace elements investigated here, only the copper concentration was significantly correlated with the serum CRP level (ρ = 0.41, *p* < 0.05). Changes in CRP concentration have previously been reported to be associated with decreased serum zinc concentrations and increased serum copper concentrations [6,22]. The association of elevated serum copper with elevated CRP observed in the present study may reflect nonspecific increases in the serum concentrations of copper-binding proteins, such as ceruloplasmin, during the acute-phase response to tuberculosis infection [6]. In the present study, serum copper concentrations were correlated with treatment outcomes as assessed after one month of standard antituberculosis therapy; specifically, culture-positive patients tended to have higher serum copper concentrations compared with culture-negative patients (*p* < 0.05). This finding is comparable to that of a previous study performed in Canada [21]. Considering the positive correlation between copper concentrations and CRP levels, increased copper concentrations could reflect host inflammation in response to live tuberculosis bacteria. Thus, serum copper and CRP concentrations may be important parameters for evaluating the persistence of nonconversion after one month of tuberculosis treatment [21].

Cobalt is required for the biosynthesis of vitamin B12 and is also an essential micronutrient for both *Mycobacterium tuberculosis* and its host [20]. A recent study reported a novel antituberculosis treatment that employed metal-based drugs by utilizing coordination complexes of copper and cobalt with the pyrophosphate ligand. This study reported notable selectivity and marked potency against *M. tuberculosis,* which implicates these metals as important for controlling tuberculosis infection [23]. However, our knowledge of the role of cobalt in tuberculosis is incomplete [20]. In the present study, serum cobalt concentrations were not significantly different between patients with tuberculosis and control patients. Moreover, no significant relationship was observed between cobalt concentration and the clinical outcome after antituberculosis treatment. Future studies are needed to define the role of cobalt in tuberculosis pathogenesis and to elucidate its potential antimycobacterial effects.

Interestingly, we observed a number of differences in trace element concentrations and demographic characteristics between patients with tuberculosis and noninfected control patients. For instance, BMI and total cholesterol were significantly lower in patients with tuberculosis compared with control patients, while the serum albumin levels of the two groups were not significantly different. Thus, total serum cholesterol appears to be a more sensitive parameter for assessing trace element concentrations in serum than serum albumin. A previous study in another Asian population (China) reported no significant differences in cholesterol concentration between patients with tuberculosis and control patients, although a significant difference in albumin level was observed [24]. This difference could be explained by the smaller sample size, which resulted in a lack of statistical significance. Further studies are needed to assess whether albumin or total cholesterol is a better indicator of trace element concentrations in the serum in patients with tuberculosis.

This study has several strengths. Firstly, this study was prospectively designed. Secondly, it is the first study to assess trace element concentrations in the serum in an ethnically homogeneous population of Korean adults. Thirdly, the study sample sizes of the case and control arms were the largest than ever reported in the literature. Finally, trace element concentrations were analyzed using a state-of-the-art ICP-MS method by which multiple elements can be screened simultaneously and with high sensitivity.

This study also had a few limitations. For instance, it was a single-center study and no information was collected regarding dietary supplementation of trace elements. Moreover, the trace element concentrations were not determined before antituberculosis treatment, thus precluding the comparison and assessment of changes in trace element concentrations and their effects on clinical outcomes. However, the current study is valuable because it is the first to assess trace element concentrations in the serum in a Korean population. It is also the first to examine potential associations between trace element concentrations and clinical outcomes after antituberculosis treatment, which will potentially inform future research. 

## 5. Conclusions

In conclusion, Korean patients with tuberculosis have altered profiles of serum trace element concentrations. Future research is needed to definitively determine whether these trace element concentrations impact clinical outcomes and to establish whether routinely providing supplements will result in improved tuberculosis treatment outcomes and/or life quality.
